# Cocaine Exposure Modulates Perineuronal Nets and Synaptic Excitability of Fast-Spiking Interneurons in the Medial Prefrontal Cortex

**DOI:** 10.1523/ENEURO.0221-18.2018

**Published:** 2018-10-04

**Authors:** Megan L. Slaker, Emily T. Jorgensen, Deborah M. Hegarty, Xinyue Liu, Yan Kong, Fuming Zhang, Robert J. Linhardt, Travis E. Brown, Sue A. Aicher, Barbara A. Sorg

**Affiliations:** 1 Department of Integrative Physiology and Neuroscience, Translational Addiction Research Center, Washington State University, Vancouver, Washington 98686; 2 Department of Physiology and Pharmacology, Oregon Health & Science University, Portland, Oregon 97239; 3 School of Pharmacy and Department of Neuroscience, University of Wyoming, Laramie, Wyoming 82071; 4 Department of Chemistry and Chemical Biology, Center for Biotechnology and Interdisciplinary Studies, Rensselaer Polytechnic Institute, Troy, New York 12180

**Keywords:** cocaine, interneurons, parvalbumin, perineuronal nets, prefrontal cortex

## Abstract

We previously reported that perineuronal nets (PNNs) are required for cocaine-associated memories. Perineuronal nets are extracellular matrix that primarily surrounds parvalbumin (PV)-containing, GABAergic fast-spiking interneurons (FSIs) in the medial prefrontal cortex (mPFC). Here we measured the impact of acute (1 d) or repeated (5 d) cocaine exposure on PNNs and PV cells within the prelimbic and infralimbic regions of the mPFC. Adult rats were exposed to 1 or 5 d of cocaine and stained for PNNs (using *Wisteria floribunda* agglutinin) and PV intensity 2 or 24 h later. In the prelimbic and infralimbic PFC, PNN staining intensity decreased 2 h after 1 d of cocaine exposure but increased after 5 d of cocaine exposure. Cocaine also produced changes in PV intensity, which generally lagged behind that of PNNs. In the prelimbic PFC, both 1 and 5 d of cocaine exposure increased GAD65/67 puncta near PNN-surrounded PV cells, with an increase in the GAD65/67-to-VGluT1 puncta ratio after 5 d of cocaine exposure. In the prelimbic PFC, slice electrophysiology studies in FSIs surrounded by PNNs revealed that both 1 and 5 d of cocaine exposure reduced the number of action potentials 2 h later. Synaptic changes demonstrated that 5 d of cocaine exposure increased the inhibition of FSIs, potentially reducing the inhibition of pyramidal neurons and contributing to their hyperexcitability during relapse behavior. These early and rapid responses to cocaine may alter the network stability of PV FSIs that partially mediate the persistent and chronic nature of drug addiction.

## Significance Statement

Parvalbumin (PV)-containing fast-spiking interneurons (FSIs) control the inhibitory/excitatory balance in the adult CNS, and the majority of these are surrounded by perineuronal nets (PNNs). The mPFC is critical to relapse in cocaine addiction, yet few studies have focused on the impact of cocaine exposure on PV interneurons that profoundly control the output of the mPFC. Our studies highlight the impact of cocaine on PV and PNN levels and the intrinsic and synaptic properties of PNN-surrounded FSIs in the mPFC. These findings point to a key upstream mechanism by which FSIs may contribute to the consistently observed increase in mPFC excitatory output of pyramidal neurons that contribute to cocaine reinstatement, and they have broader implications for understanding how PNN-surrounded neurons may control aberrant learning processes involved in addiction.

## Introduction

Pyramidal neurons in the medial prefrontal cortex (mPFC) that project to the nucleus accumbens control cocaine-seeking behavior in rodent models of addiction (McFarland and Kalivas, 2001; McLaughlin and See, 2003; McFarland et al., 2004; Ma et al., 2014). However, surprisingly few studies (Kroener and Lavin, 2010; Campanac and Hoffman, 2013) have examined the impact of cocaine on fast-spiking GABAergic interneurons, which contain parvalbumin (PV; Kawaguchi and Kubota, 1993). PV neurons profoundly control the output of these mPFC pyramidal neurons, and in the mPFC, PV fast-spiking interneurons (FSIs) contain D_1_ and D_2_ dopamine receptors (Le Moine and Gaspar, 1998) and synapse directly onto layer V pyramidal neurons to control their output (Lee et al., 2014).

Approximately 60-80% of PV cells in the cortex are enwrapped by perineuronal nets (PNNs; Brückner et al., 1993; Slaker et al., 2015), which are aggregations of extracellular matrix (ECM) molecules that appear during development in an experience-dependent manner. The maturation of PV cells with PNNs coincides with the closure of developmental critical periods and PV network stabilization (Pizzorusso et al., 2002; Balmer et al., 2009; Gogolla et al., 2009; Kwok et al., 2011). Several studies have demonstrated an important contribution of PNNs to learning and memory (Balmer et al., 2009; Fawcett, 2009; Gogolla et al., 2009; Romberg et al., 2013; Banerjee et al., 2017; Foscarin et al., 2017). We previously found that the consolidation and reconsolidation of cocaine-associated memories were dependent on intact PNNs in the mPFC (Slaker et al., 2015), and others have reported a role for PNNs in drug-related behaviors (Van den Oever et al., 2010b; Xue et al., 2014; Vazquez-Sanroman et al., 2015b).

The findings that the removal of PNNs alters learning and memory strongly suggest that PNNs themselves may be a target for stimuli that induce learning and memory, including drugs of abuse. Even during adulthood, PNNs are dynamic structures modulated by experience that changes the intensity of PNN staining. For example, exposure to environmental enrichment in rodents decreases the intensity of PNN staining within the somatosensory cortex, motor cortex, and cerebellum (Foscarin et al., 2011; Madinier et al., 2014). The intensity of PNNs [as labeled by *Wisteria floribunda* agglutinin (WFA)] is commonly used as an indirect measure of their developmental maturity, with dim staining representing an immature PNN and bright staining representing a mature PNN (Foscarin et al., 2011; Wang and Fawcett, 2012). Dynamic changes in PV intensity also occur after learning and memory, and are associated with changes in PV network activity that powerfully controls the output of neurons embedded within the network (Donato et al., 2013; Favuzzi et al., 2017)

Here we defined the early impacts of acute (1 d) and repeated (5 d) cocaine exposure dynamic changes on the intensity of PNNs and PV and on electrical signaling in PNN-surrounded FSIs in the mPFC of adult rats. Early changes in these neurons may well contribute to the cocaine-induced hyperexcitability of mPFC pyramidal neurons reported by us and several others (Dong et al., 2005; Nasif et al., 2005; Huang et al., 2007; Hearing et al., 2013; Slaker et al., 2015), which promotes reinstatement behavior. In the current study, we determined the extent to which acute and repeated cocaine exposure altered the intensity of PNNs and PV as well as functional changes in FSIs surrounded by PNNs. We found that acute cocaine exposure decreased PNNs and PV intensity, while repeated cocaine exposure increased PNNs and PV intensity in the prelimbic PFC, suggesting that acute cocaine exposure shifted PV cells to a less mature state, while repeated cocaine exposure shifted these cells to a more mature state. Repeated cocaine exposure decreased the excitability of PV FSIs, consistent with an increase in the inhibitory/excitatory ratio of puncta in these cells, and it increased miniature IPSCs (mIPSC) frequency and amplitude. Altogether, these changes may significantly contribute to the hyperexcitability of pyramidal neurons in the prelimbic PFC that contribute to drug reinstatement.

## Materials and Methods

### Animals

Adult male Sprague Dawley rats obtained from Simonsen Laboratories were used in these studies. A total of 127 rats were used [56 for WFA/PV intensity analyses (a subset of 16 rats was used for puncta analysis), 16 for chondroitin sulfate proteoglycan (CSPG) analyses, and 55 for electrophysiological recordings (29 for intrinsic recordings and 26 for synaptic recordings]. Rats weighed 330.1 ± 2.5 g (mean ± SEM) at the start of each experiment. All animals were singly housed in a temperature- and humidity-controlled room with a 12 h light/dark cycle in which lights were on at 7:00 A.M. or 7:00 P.M. Previous work has demonstrated no changes in early cocaine sensitization at these times (Sleipness et al., 2005). Food and water were available *ad libitum* throughout the experiment, except during behavioral testing. All experiments were approved by the Washington State University and the University of Wyoming Institutional Animal Care and Use Committees and were conducted according to the National Institutes of Health *Guide for the Care and Use of Laboratory Animals*. All efforts were made to reduce the number of animals and to minimize pain and suffering.

### Drugs

Cocaine hydrochloride was a gift from the National Institute on Drug Abuse. The cocaine salt was dissolved in sterile saline as the weight of the salt to a final concentration of 15 mg/ml. Cocaine and saline were administered intraperitoneally at a concentration of 1 ml/kg.

### Materials for chondroitin sulfate proteoglycan analysis

Unsaturated disaccharide standards of chondroitin sulfate (CS; ΔUA-GalNAc; ΔUA-GalNAc4S; ΔUA-GalNAc6S; ΔUA2S-GalNAc; ΔUA2S-Gal-NAc4S; ΔUA2S-GalNAc6S; ΔUA-GalNAc4S6S; ΔUA2S-GalNAc4S6S), unsaturated disaccharide standards of heparin sulfate (HS; ΔUA-GlcNAc; ΔUA-GlcNS; ΔUA-GlcNAc6S; ΔUA2S-GlcNAc; ΔUA2S-GlcNS; ΔUA-GlcNS6S; ΔUA2S-GlcNAc6S; ΔUA2S-GlcNS6S), and unsaturated disaccharide standard of hyaluronic acid (HA; ΔUA-GlcNAc), where ΔUA is 4-deoxy-α-L-*threo*-hex-4-enopyranosyluronic acid, were purchased from Iduron. Actinase E was obtained from Kaken Biochemicals. Chondroitin lyase ABC from *Proteus vulgaris* was expressed in the laboratory of R.J.L. Recombinant flavobacterial heparin lyases I, II, and III were expressed in the R.J.L. laboratory using *Escherichia coli* strains provided by Jian Liu (College of Pharmacy, University of North Carolina, Chapel Hill, NC). 2-aminoacridone (AMAC) and sodium cyanoborohydride (NaCNBH_3_) were obtained from Sigma-Aldrich. All other chemicals were of HPLC grade. Vivapure Q Mini H strong anion exchange spin columns were from Sartorius.

### Cocaine exposure

A three-chamber apparatus (total dimensions, 68 × 21 × 21 cm) was used to assess locomotor activity (Med Associates). Animals were allowed access to the entire apparatus, and locomotor activity was recorded automatically with infrared photocell beams.

Rats were gently handled for 5 d before the start of the experiment. On the first day of the experiment, rats were given a 15 min period for habituation to the apparatus to limit novelty-induced locomotor effects on future testing days. Rats were assigned to the cocaine or saline group by counterbalancing locomotor activity during the habituation day. On subsequent days, rats were given a dose of saline (1 mL/kg, i.p.) or cocaine (15 mg/kg, i.p.) and immediately placed in the apparatus for 30 min with locomotor activity reported the first 15 min.

### Tissue collection

For immunohistochemistry, 2 or 24 h following the last injection, rats were perfused intracardially with 4% paraformaldehyde (PFA) in PBS. The brains were removed and stored overnight in 4% PFA at 4˚C. The following day, brains were moved to a 20% sucrose solution, and 24 h later were frozen with powdered dry ice and stored at −80 ˚C until analysis. For mass spectrometry, 2 h following the last injection, rats were rapidly decapitated, and mPFC tissue containing primarily the prelimbic PFC was dissected and flash frozen on dry ice in a microcentrifuge tube. Tissue was stored at −80˚C and shipped on dry ice.

### Immunohistochemistry and imaging

These studies were conducted as previously described (Slaker et al., 2016). Anatomic landmarks were used to determine representative caudal and rostral sections of prelimbic cortex that were within bregma approximately +3.2 to +4.2 mm (Paxinos, 1998). Serial coronal brain sections were cut at 30 µm using a freezing microtome. Sections were maintained in a storage buffer solution until immunohistochemistry was performed. To assess PV and WFA staining, free-floating sections were washed three times for 5 min in PBS and then quenched in 50% ethanol for 30 min. After a set of three 5 min washes in PBS, the sections were placed in 3% goat blocking serum (Vector Laboratories) for 1 h. Tissue was incubated at 4˚C on a rocker overnight with an antibody against PV (1:1000; catalog #MAB1572, Millipore; RRID:AB_2174013) in PBS containing 2% goat serum. After three 10 min washes, tissue was incubated for 2 h with secondary goat anti-mouse Alexa Fluor-594 antibody (1:500; catalog #R37121, Thermo Fisher Scientific; RRID:AB_2556549). After three 10 min washes in PBS, tissue was incubated for 2 h with fluorescein-conjugated WFA (1:500; catalog #FL-1351, Vector Laboratories; RRID:AB_2336875). Tissue was washed three more times for 10 min and then mounted on Frost Plus slides in diluted PBS with Triton X-100 (0.15× PBS, 0.24% Triton X-100). Slides were stored flat until dry (at least 24 h) at 4˚C. The slides were coverslipped with ProLong Gold (Life Technologies) and stored flat at 4˚C until the time of imaging. Images of the prelimbic and infralimbic PFC were taken using a Leica SP8 Laser-Scanning Confocal Microscope with the Leica Application Suite. An HCX PL Apo CS, dry, 20× objective with 0.70 numerical aperture (NA) was used for all images. WFA-bound fluorescein was excited using a 488 laser, and a photomultiplier tube detected emission photons within the range of 495–545 nm. Alexa Fluor-594 was excited using a 561 laser, and a photomultiplier tube detected emission photons within the range of 585–645 nm. Images were taken through a *z*-plane (9 µm) at the center of the tissue containing 20 stacks within each region. Gain, offset, laser intensity, zoom, and pinhole were kept constant for all images. Sequences of the raw images from the *z*-stack were exported and projected into a sum slices image using ImageJ software (NIH).

### Quantification and statistical analysis

WFA and PV intensity were quantified using ImageJ software (RRID:SCR_003070) as previously described (Slaker et al., 2016). Briefly, background subtraction from each projection image was conducted by first using the Rolling Ball Radius function and then determining 2 SDs above the mean within a region of the image containing no visible PNNs. Each visible PNN (surrounding at least two-thirds of the underlying cell body) in the image was assigned as a region of interest, including the cell body and proximal dendrites. The average intensity for each region of interest (each PNN) was calculated and recorded. All intensity values were normalized to the average intensity value from the control group (saline) for each region. To assess correlations between WFA and PV staining intensity, raw PV intensity was grouped together based on bins of 10 arbitrary units (AUs) and then within each bin the corresponding raw intensity of WFA (in AUs) from the same cell was averaged. All measurements were made by an experimenter blinded to the treatment conditions.

### Puncta labeling and imaging

Immunohistochemical methods were similar to those previously described (Hegarty et al., 2010, 2014). Solutions were prepared in either 0.1 m phosphate buffer at pH 7.4 (PB) or 0.1 m Tris-buffered saline at pH 7.6 (TS). Tissue sections were first rinsed in PB then incubated in 1% sodium borohydride in PB for 30 min to reduce background. After rinses in PB and TS, sections were incubated in 0.5% bovine serum albumin (BSA) in TS for 30 min and then placed in a primary antibody cocktail made in 0.1% BSA and 0.25% Triton X-100 in TS for 2 nights at 4°C. The primary antibody cocktail consisted of goat anti-glutamic acid decarboxylase 65/67 (GAD65/67; 1:100; catalog #sc-7513, Santa Cruz Biotechnology; RRID:AB_2107745), rabbit anti-parvalbumin (1:1000, catalog #NB120-11427, Novus Biologicals; RRID:AB_791498), and guinea pig anti-vesicular glutamate transporter 1 (VGluT1; 1:5000; catalog #AB5905, EMD Millipore; RRID:AB_2301751). After 40 h of primary antibody incubation, tissue sections were rinsed in TS and then incubated with a cocktail of fluorescently labeled secondary antibodies for 2 h, light protected, at room temperature. The secondary antibody cocktail consisted of Alexa Fluor-488 donkey anti-goat (1:800; catalog #A11055, Thermo Fisher Scientific; RRID:AB_2534102), Alexa Fluor-546 donkey anti-rabbit (1:800, catalog #A10040, Thermo Fisher Scientific; RRID:AB_2534016), and Alexa Fluor 647 donkey anti-guinea pig (1:800, catalog #706-605-148, Jackson ImmunoResearch; RRID:AB_2340476). Tissue sections were rinsed again in TS and then incubated in biotinylated WFA (1:50; catalog #B1355, Vector Laboratories; RRID:AB_2336874) for 2 h at room temperature. Following TS rinses, tissue sections were incubated for 3 h at room temperature in Alexa Fluor-405-conjugated streptavidin (6.25 µg/ml; catalog #S32351, Thermo Fisher Scientific). Finally, tissue sections were rinsed in TS followed by PB before being mounted with 0.05 m PB onto gelatin-coated slides to dry. Slides were coverslipped with Prolong Gold Antifade Mountant (Thermo Fisher Scientific) and light protected until imaging.

The anatomic landmarks used were those described above for PV and WFA labeling. To determine representative caudal and rostral sections of prelimbic cortex that were within +3.5 to +4.2 mm from bregma (Paxinos, 1998), two high-magnification images were taken at each level of the prelimbic cortex (2 images/level × 2 levels/animal = 4 images/animal). Images were captured on a Zeiss LSM 780 confocal microscope with a 63× 1.4 NA Plan-Apochromat objective (Carl Zeiss MicroImaging) using the single-pass, multitracking format at a 1024 × 1024 pixel resolution. Optical sectioning produced *z*-stacks bounded by the extent of fluorescent immunolabeling throughout the thickness of each section. Using Zen software (Carl Zeiss; RRID:SCR_013672), PV neurons in each confocal stack were identified and assessed for the presence of a nucleus and whether the entire neuron was within the boundaries of the field of view; only these PV neurons were included in the analysis. The optical slice through the nucleus at which the ellipsoidal minor axis length of each PV neuron reached its maximum was determined. A *z*-stack of that optical slice plus one optical slice above and one below was created, resulting in a 1.15 µm *z*-stack through the middle of each PV neuron; these subset *z*-stacks were used for puncta apposition analysis.

Image analysis of GABAergic and glutamatergic appositions onto PV-labeled neurons was performed using Imaris 8.0 software (BitPlane USA; RRID:SCR_007370) on an off-line workstation in the Advanced Light Microscopy Core at Oregon Health & Science University by a blinded observer. For each PV neuron, the manual setting of the *Surfaces* segmentation tool was used to trace the outline of the PV neuron in all three optical slices, and a surface was created. To limit our analyses to the area immediately surrounding each PV neuron, we used the *Distance Transform* function followed by the automated *Surfaces* segmentation tool to create another surface 1.5 µm away from the PV neuron surface that followed the unique contours of that PV neuron. The *Mask Channel* function was then used to examine only WFA, GAD65/67, and VGluT1 within this 1.5-µm-wide perimeter surrounding the PV neuron surface.

The presence of WFA labeling in close proximity to the PV neuron surface was assessed for each PV neuron. A PV neuron was considered to have a PNN if there was any WFA labeling around any part of the PV neuron surface as seen by the observer. GAD65/67 and VGluT1-labeled puncta were then assessed separately using the *Spots* segmentation tool. Within the *Spots* tool, the *Different Spot Sizes (Region Growing)* option was selected and initial settings included an estimated *x*–*y* diameter of 0.5 µm and an estimated *z*-plane diameter of 0.4 µm. Spots generated by Imaris from these initial settings were then thresholded using the *Classify Spots*, *Quality Filter* histogram to ensure that all labeled puncta were included and background labeling was filtered out. The spots were then thresholded using the *Spot Region, Region Threshold* histogram to ensure that the sizes of the Imaris-generated spots were good approximations of the size of the labeled puncta seen visually by the human observer. Using the *Find Spots Close to Surface Imaris XTension*, we then isolated those spots that were within 0.5 µm of the PV neuron surface. All segmented spots close to the PV neuron surface had to have a *z*-diameter of at least 0.4 µm to be considered puncta (Hegarty et al., 2010, 2014).

### Liquid chromatography-mass spectrometry

Freeze-dried brain samples were defatted with 0.5 ml of acetone for 30 min, vortexed, and dried in the hood. Each defatted sample was digested in 0.2 ml of Actinase E (2.5 mg/ml) at 55°C until all of the tissue was dissolved (∼48 h). After centrifugation at 12,000 rpm, each supernatant was collected and purified by Mini Q spin columns. Protein contaminates were washed out three times with 0.2 ml of 0.2 m NaCl, and the glycosoaminoglycans (GAGs) were eluted from a SAX spin column using 0.5 ml of 16% NaCl. Samples eluted from a Mini Q spin column were desalted by passing through a 3 kDa molecular weight cutoff spin column and washed twice with distilled water. The desalted samples were dissolved in 150 µl of digestion buffer (50 mm ammonium acetate containing 2 mm calcium chloride adjusted to pH 7.0) in filtration units. Recombinant heparin lyases I, II, and II (optimum pH 7.0–7.5) and recombinant chondroitin lyase ABC (10 mU each, optimum pH 7.4) were added to each sample and mixed well. The samples were all placed in a water bath at 37°C for 2 h. The enzymatic digestion was terminated by removing the enzymes by centrifugation. The filter unit was washed twice with 100 µl of distilled water and the filtrates containing the disaccharide products were lyophilized. The dried samples were AMAC labeled by the addition of 10 µl of 0.1 m AMAC in DMSO/acetic acid (17/3, v/v) and incubation at room temperature for 10 min, followed by the addition of 10 µl of 1 m aqueous NaBH_3_CN and incubation for 1 h at 45°C. A mixture containing all 17-disaccharide standards prepared at 6.25 ng/μl was similarly AMAC labeled and used for each run as an external standard. After the AMAC-labeling reaction, the samples were centrifuged and each supernatant was recovered. Liquid chromatography (LC) was performed on an Agilent 1200 LC system at 45°C using an Agilent Poroshell 120 ECC18 (2.7 μm, 3.0 × 50 mm) column. Mobile phase A was 50 mm ammonium acetate aqueous solution, and mobile phase B was methanol. The mobile phase passed through the column at a flow rate of 300 µl/min. The gradients were as follows: 0–10 min, 5–45% B; 10–10.2 min, 45–100% B; 10.2–14 min, 100% B; and 14–22 min, 100–5% B. The injection volume is 5 µl. A triple-quadrupole mass spectrometry system equipped with as an electrospray ionization source (Thermo Fisher Scientific) was used a detector. The on-line mass spectrometric analysis was performed in multiple reaction monitoring mode. Mass spectrometry parameters were as follows: negative ionization mode with spray voltage of 3000 V, a vaporizer temperature of 300°C, and a capillary temperature of 270°C.

### Whole-cell patch-clamp electrophysiology

For whole-cell patch clamp, rats were anesthetized with isoflurane 2 h after the last saline or cocaine injection followed by intracardial perfusion with a recovery solution oxygenated with 95% O_2_-5% CO_2_ at ice-cold temperatures. The composition of the recovery solution was as follows (in mm): 93 NMDG, 2.5 KCl, 1.2 NaH_2_PO_4_, 30 NaHCO_3_, 20 HEPES, 25 glucose, 4 sodium ascorbate, 2 thiourea, 3 sodium pyruvate, 10 MgSO_4_(H_2_O)_7_, and 0.5 CaCl_2_(H_2_O_2_), and HCl was added until pH was 7.3–7.4 with an osmolarity of 300–310 mOsm. Following perfusion, rats were decapitated, and coronal slices (300 µm) containing the prelimbic PFC (∼3.2–3.7 mm from bregma) were sliced as previously described (Slaker et al., 2015) in an ice-cold recovery solution using a vibratome (model VT1200S, Leica). Before recording, slices were incubated for 1 h in a room temperature holding solution oxygenated with 95% O_2_-5% CO_2_. The composition of the holding solution was as follows (in mm): 92 NaCl, 2.5 KCl, 1.2 NaH_2_PO_4_, 30 NaHCO_3_, 20 HEPES, 25 glucose, 4 sodium ascorbate, 2 thiourea, 3 sodium pyruvate, 2 MgSO_4_(H_2_O)_7_, and 2 CaCl_2_(H_2_O_2_), and 2 m NaOH was added until pH reached 7.3–7.4 and osmolarity was 300–310 mOsm. Immediately before recording, slices were incubated in a room temperature holding solution containing WFA (1 µg/ml) for 5 min to stain for PNNs. Each slice was transferred to the recording chamber and fixed to the bottom of the chamber using a platinum harp. The recording chamber was perfused constantly at 31.0°C at a rate of 4–7 mL/min aCSF. The aCSF composition was as follows (in mm): 119 NaCl, 2.5 KCl, 1 NaH_2_PO_4_, 26 NaHCO_3_, 11 dextrose, 1.3 MgSO_4_(H_2_O)_7_, and 2.5 CaCl_2_(H_2_O)_2_. CellSens software (Olympus) was used to identify fluorescing cells so that neurons surrounded by PNNs could be patched. Patching pipettes were pulled from borosilicate capillary tubing (Sutter Instruments), and the electrode resistance was typically 4–7 mΩ. Cells were current clamped at −70 mV and 10 current steps were injected starting at −100 pA and ending at 800 pA. Elicited action potentials were recorded, counted, and analyzed using pClamp version 10.3 (Clampfit, Molecular Devices).

Mini Analysis (Synaptosoft) was used to measure miniature amplitudes and frequencies. To record mIPSCs, perfusing aCSF bath contained the following: 6,7-dinitroquinoxaline-2,3-dione (10 μm) and strychnine (1 μm), and tetrodotoxin (1 μm) to block AMPA, sodium, and glycine and sodium receptors/channels, respectively. The composition of the intracellular solution was as follows (in mm): 117 CsCl, 2.8 NaCl, 5 MgCl_2_, 20 HEPES, 2 Mg^2+^ATP, 0.3 Na^2+^GTP, and 0.6 EGTA, and sucrose to bring osmolarity to 275–280 mOsm and pH to ∼7.25. To record miniature EPSCs (mEPSCs), aCSF contained picrotoxin (100 μm) and tetrodotoxin (1 μm). Patch pipettes were filled with the following (in mm): 125 KCL, 2.8 NaCl, 2 MgCl_2_, 2 ATP-Na^+^, 0.3 GTP-Li^+^, 0.6 EGTA, and 10 HEPES. Cells were voltage clamped at −70 mV, and input resistance and series resistance were monitored throughout the experiments. IPSCs and EPSCs were amplified and recorded using pClamp version 10.3. The Mini Analysis Program Demo (Synaptosoft) was used to measure the peak mIPSC amplitudes or peak mEPSC amplitudes.

### Statistical analysis

All statistical tests were conducted using Prism 6 software (GraphPad Software). A two-way ANOVA with repeated measures was used across days to assess changes in locomotor activity following cocaine exposure and for determining the number of action potentials across current steps. In the case of a significant interaction, a Sidak’s test was used for multiple comparisons. Two-tailed, Student’s *t* tests were used to compare the number of WFA^+^/PV^+^ cells between treatment conditions (Table 1). A Kolmogorov–Smirnoff nonparametric test for distribution was used to compare the intensity of WFA and PV among treatment conditions and also for miniature analyses. A Mann–Whitney *U* test was used for puncta analysis. Correlation between WFA and PV intensity and between cocaine-induced locomotor activity and WFA/PV intensity were performed using linear regression analysis. Significance was determined at *p* < 0.05.

**Table 1.** Number of PV cells surrounded by WFA in prelimbic and infralimbic PFC after 1 or 5 d of saline or cocaine

Group	Drug	Region	PV^+^/WFA^+^
1 d 2 h	Sal	Prelimbic	88 ± 14
	Coc		77 ± 8
	Sal	Infralimbic	61 ± 6
	Coc		51 ± 7
5 d 2 h	Sal	Prelimbic	80 ± 12
	Coc		70 ± 10
	Sal	Infralimbic	61 ± 8
	Coc		66 ± 8
1 d 24 h	Sal	Prelimbic	89 ± 18
	Coc		91 ± 21
	Sal	Infralimbic	45 ± 5
	Coc		43 ± 11
5 d 24 h	Sal	Prelimbic	102 ± 16
	Coc		108 ± 14
	Sal	Infralimbic	67 ± 8
	Coc		49 ± 9

Coc, Cocaine; Sal, saline.

## Results

### Cocaine exposure induces locomotor activity

**Figure 1.**
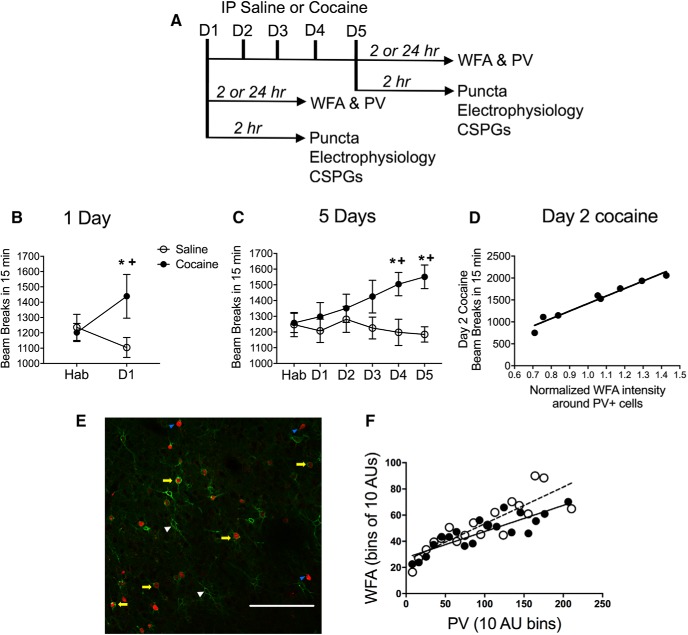
Cocaine exposure increases locomotor activity, and PV intensity is correlated with WFA intensity. Data are the mean ± SEM. ***A***, Timeline of experiment. ***B***, Infrared photocell beam breaks the first 15 min after saline or cocaine injection (15 mg/kg, i.p.). A single cocaine exposure increased the locomotor response compared with the habituation (Hab) day. ***C***, Repeated cocaine increased locomotor activity compared with their Hab day and with saline controls on day 4 (D4) and D5. ***D***, Significant positive correlation between WFA staining intensity in WFA^+^/PV^+^ cells and cocaine-induced locomotor activity on day 2 when intensity was examined 2 h after 5 d of cocaine exposure (Fig. 1-1, available at https://doi.org/10.1523/ENEURO.0221-18.2018.f1-1, for all correlations). ***E***, Representative photomicrograph of PV cells (red) with (yellow arrows) and without (blue arrows) WFA (green). WFA was also observed surrounding non-PV cells (white arrows). Scale bar, 100 µm. ***F***, Significant positive correlation between WFA and PV intensity in rats 24 h after 5 d of saline (open circles) or cocaine (closed circles) exposure. For ***B*** and ***C***: *p < 0.05, compared with saline group; +*p* < 0.05, compared with Hab group.

Figure 1*A* shows the timeline for all experiments. We show the locomotor activity of every rat used in these studies after acute and repeated saline or cocaine exposure, the latter of which produces behavioral sensitization (Robinson and Berridge, 1993). Figure 1*B* indicates that locomotor activity increased following acute cocaine injection compared with the habituation day. There was a significant treatment × time interaction (*F*_(1,63)_ = 8.96, *p* = 0.0039; *N* = 31–34/group). Figure 1*C* shows the locomotor response each day after 5 d of cocaine exposure. There was a trend toward a main effect of treatment (*F*_(1,60)_ = 3.88, *p* = 0.053; *N* = 30–32/group) and a significant treatment × time interaction (*F*_(5,300)_ = 3.52, *p* = 0.004). Sidak’s *post hoc* analysis indicated that, compared with the habituation day and the saline controls, cocaine elevated locomotor activity on days 4 and 5. In addition, activity was higher on day 5 of cocaine exposure compared with day 1 of cocaine exposure, indicating that locomotor sensitization had occurred.

10.1523/ENEURO.0221-18.2018.f1-1 Figure 1-1 Table showing significant correlations between cocaine-induced locomotor activity and WFA or PV intensity in single- and double-labeled cells in the prelimbic and infralimbic PFC. Download Figure 1-1, DOC file.

### Cocaine exposure alters intensity of WFA and PV staining within the prelimbic and infralimbic PFC

WFA is a common marker for PNNs (Härtig et al., 1992) and has been used as an indirect measure of maturity of PNNs (Foscarin et al., 2011; Cabungcal et al., 2013a; Chen et al., 2015; Vazquez-Sanroman et al., 2015a). To test whether PNNs were altered by cocaine exposure, we assessed both the intensity and number of PV-stained cells with or without WFA surrounding these cells after 1 or 5 d of saline or cocaine exposure. Linear regression analyses were conducted to determine whether there was a correlation between cocaine-induced locomotor activity and WFA and/or PV staining intensity. All significant correlations are shown in the table in Figure 1-1 (available at https://doi.org/10.1523/ENEURO.0221-18.2018.f1-1). The strongest correlations were found in the prelimbic PFC between WFA^+^/PV^+^ cells 2 h after 5 d of cocaine exposure and cocaine-induced locomotor behavior on day 2 (*p* < 0.0001) and day 3 (*p* = 0.0032; Fig. 1*D*, day 2 results). While there was no correlation with day 1 cocaine-induced behavior (*p* = 0.4998), behavior on days 2 and 3 was strongly correlated, behavior on day 4 was not significant (*p* = 0.1793), and behavior on day 5 was nearly significant (*p* = 0.0633). In the same animals, we also found a correlation between the intensity of single-labeled WFA cells (WFA^+^/PV^−^ cells) and behavior on day 2 (*p* = 0.0138) and day 3 (*p* = 0.0430). This finding suggests that an increase in WFA around PV cells may occur early and that the increase may be sustained up to 5 d later, when PNNs were measured. This effect appeared only at 2 h and did not persist in animals assessed 24 h later. Other correlations found in the prelimbic PFC were from rats that were exposed to cocaine for 5 d and assessed 24 h later. In these rats, there was a weak negative correlation in single-labeled WFA cells with day 1 cocaine-induced locomotor activity (*p* = 0.0462). In this same group of rats, there was also a negative correlation in the prelimbic PFC between PV staining (PV^+^/WFA^+^ and PV^+^/WFA^−^ cells) with day 1 cocaine-induced locomotor activity (*p* = 0.0124 and *p* = 0.0364, respectively). None of the correlations we observed in the prelimbic PFC were observed in the infralimbic PFC. In the infralimbic PFC, we found a negative correlation after acute cocaine exposure between staining with WFA in both WFA^+^/PV^+^ and WFA^+^/PV^−^ cells with locomotor activity 24 h later (*p* = 0.0146 and *p* = 0.0267, respectively). There was also a positive correlation between WFA staining in WFA^+^/PV^−^ cells and locomotor activity 2 h after day 5 of cocaine exposure (*p* = 0.0125). In addition, we found a positive correlation between the staining with WFA in both WFA^+^/PV^+^ and WFA^+^/PV^−^ cells and 24 h after day 3 of cocaine-induced activity (*p* = 0.0363 and *p* = 0.0170, respectively). There were no correlations between PV staining and cocaine-induced activity in the infralimbic PFC.

Figure 1*E* is a photomicrograph of PV single-labeled cells and PV double-labeled cells with WFA to stain for PNNs. We also examined whether there was a correlation between the intensity of PV and WFA staining in the prelimbic and infralimbic PFC. Previous studies have shown that weakly stained PV cells tend to lack PNNs, and that the digestion of PNNs with chondroitinase ABC decreases PV levels (Yamada et al., 2015), suggesting that the expression of PNNs impacts the expression of PV or vice versa. In nearly all cases in both the prelimbic and infralimbic PFC, we found a positive correlation between the intensity of WFA and PV within the same cells; an example of this correlation for the prelimbic PFC 24 h after 5 d of treatment is shown in Figure 1*E* (saline *R* ^2^ = 0.74, *p* < 0.0001; cocaine *R* ^2^ = 0.73, *p* < 0.0001).

**Figure 2.**
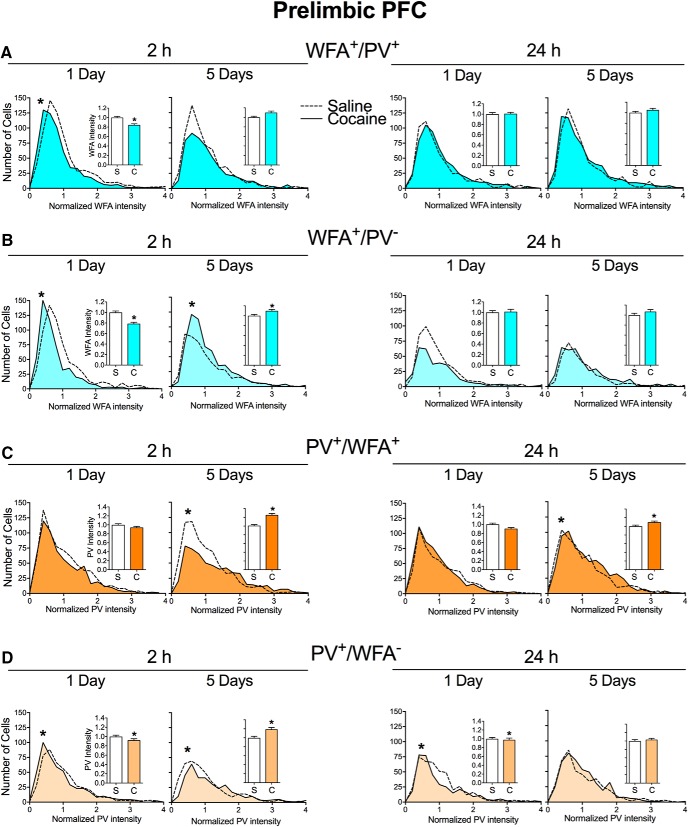
Cocaine exposure alters the intensity of WFA and PV in the prelimbic PFC. ***A***, ***B***, Intensity of WFA surrounding PV cells (WFA^+^/PV^+^ cells; ***A***) and intensity of WFA surrounding all non-PV cells (WFA^+^/PV^−^ cells; ***B***) 2 or 24 h after acute (1 d) or repeated (5 d) saline (S) or cocaine (C) exposure in the prelimbic PFC. ***C***, ***D***, Intensity of PV cells that are surrounded by WFA (PV^+^/WFA^+^ cells; ***C***) and intensity of PV^+^/WFA^−^ cells (***D***) 2 or 24 h after acute or repeated saline or cocaine. *N* = 6–8 rats/group. *p < 0.05, compared with saline group.

Figure 2 shows the distribution of the intensity of WFA and PV staining in the prelimbic PFC, with insets showing the same data represented in bar graphs for ease of visibility. Figure 2, *A* and *B*, shows the distribution of intensity of WFA staining around PV cells (WFA^+^/PV^+^ cells; Fig. 2*A*) and WFA single-labeling intensity (WFA^+^/PV^−^ cells, Fig. 2*B*). At 2 h after acute cocaine exposure, we observed a decrease in the intensity of both WFA^+^/PV^+^ cells (Fig. 2*A*; *p* = 0.0002) and WFA^+^/PV^−^ cells (Fig. 2*B*; *p* < 0.0001). In contrast, after repeated cocaine exposure, the intensity of WFA^+^/PV^−^ cells was increased (*p* = 0.0033) 2 h later. After either acute or repeated cocaine exposure, there were no differences in WFA^+^/PV^+^ or WFA^+^/PV^−^ cell intensity 24 h later (Fig. 2*A*,*B*, respectively). Thus, 1 d of cocaine exposure rapidly (2 h) decreased WFA intensity, while 5 d of cocaine exposure produced the opposite effect, indicating that PNNs in the prelimbic PFC are differentially altered by acute versus chronic exposure to cocaine.

PV cells are important regulators of learning and memory, with PV expression levels relatively low during learning and relatively high after memory consolidation (Donato et al., 2013; Favuzzi et al., 2017). Figure 2, *C* and *D*, shows the distribution of the intensity of PV staining in PV^+^/WFA^+^ cells and PV^+^/WFA^−^ cells in the prelimbic PFC. After 1 d of cocaine exposure, there was a trend toward a decrease in PV intensity in PV^+^/WFA^+^ cells 2 h later (Fig. 2*C*; *p* = 0.0792), and a small but significant decrease in PV^+^/WFA^−^ cells at this time point (Fig. 2*D*; *p* = 0.0067). These decreases were maintained 24 h later, again with a trend toward a decrease in intensity of PV^+^/WFA^+^ cells (*p* = 0.0700), and a significant decrease in intensity of PV^+^/WFA^−^ cells (*p* = 0.0164). After 5 d of cocaine exposure, PV intensity was increased 2 h later for both PV^+^/WFA^+^ cells (*p* < 0.0001) and PV^+^/WFA^−^ cells (*p* = 0.005), primarily due to a greater number of cells with less intense staining in the saline group. This increase was maintained 24 h later for PV^+^/WFA^+^ cells (*p* = 0.0137). Thus, similar to the effects of cocaine on WFA intensity, 1 and 5 d of cocaine exposure produced opposite effects on PV intensity.

**Figure 3.**
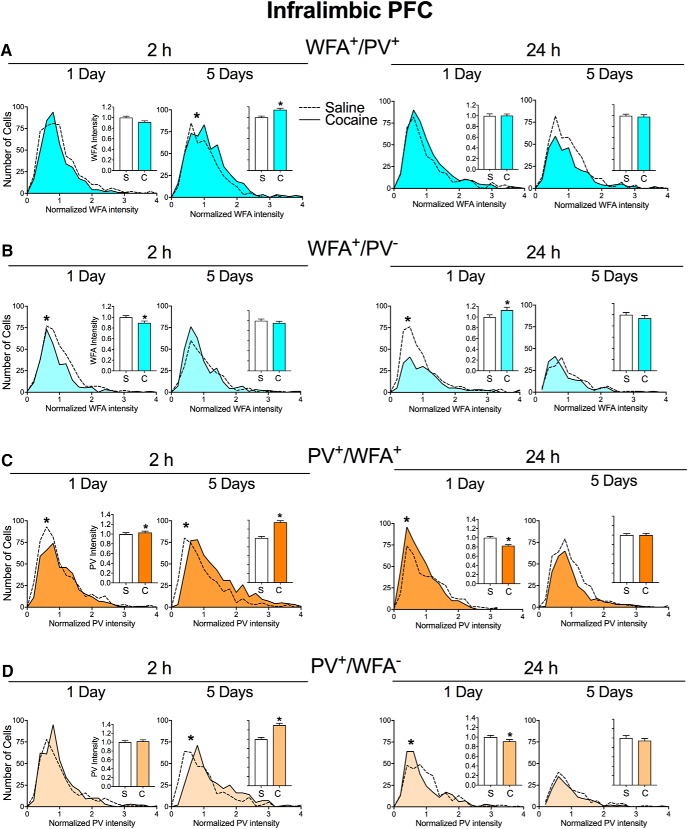
Cocaine exposure alters intensity of WFA and PV in the infralimbic PFC. ***A***, ***B***, Intensity of WFA surrounding PV cells (WFA^+^/PV^+^ cells; ***A***) and intensity of WFA surrounding all non-PV cells (WFA^+^/PV^−^ cells; ***B***) 2 or 24 h after acute (1 d) or repeated (5 d) saline (S) or cocaine (C) exposure in the infralimbic PFC. ***C***, ***D***, Intensity of PV cells that are surrounded by WFA (PV^+^/WFA^+^ cells; ***C***) and intensity of PV^+^/WFA^−^ cells PV cells (***D***) at 2 or 24 h after acute or repeated saline or cocaine exposure. *N* = 6–8 rats/group. *p < 0.05, compared to saline group.

Figure 3 shows the distribution of intensity of WFA and PV staining in the infralimbic PFC, with insets showing the same data represented in bar graphs for ease of visibility. Figure 3, *A* and *B*, shows the distribution of intensity of WFA staining around PV cells (WFA^+^/PV^+^ cells; Fig. 3*A*) and WFA single-labeling intensity (WFA^+^/PV^−^ cells; Fig. 3*B*). At 2 h after acute cocaine exposure, there was a small but significant decrease in WFA^+^/PV^−^ cells (Fig. 3*B*; *p* = 0.0036). After repeated cocaine exposure, there was an increase in WFA^+^/PV^+^ cells at this same time point. After 24 h, there was a trend toward the intensity of WFA^+^/PV^−^ cells (*p* = 0.0701) and a significant increase in WFA^+^/PV^−^ cells after acute cocaine exposure (*p* = 0.0124).

Figure 3, *C* and *D*, shows the distribution of PV intensity staining in PV^+^/WFA^+^ and PV^+^/WFA^−^ cells in the infralimbic PFC. Acute cocaine exposure slightly but significantly increased the intensity of PV^+^/WFA^+^ cells (Fig. 2*C*; *p* = 0.0392) 2 h later, while repeated cocaine exposure increased the intensity of both PV^+^/WFA^+^ cells (*p* < 0.0001) and PV^+^/WFA^−^ cells (*p* < 0.0001) at this same time point. At 24 h following acute cocaine exposure, the intensity of both PV^+^/WFA^+^ cells (*p* = 0.0002) and PV^+^/WFA^−^ cells (*p* = 0.0018) was decreased, and there were no changes 24 h after repeated cocaine exposure. Thus, changes in the infralimbic PFC were similar to those of the prelimbic PFC in most, but not all, conditions.

We also determined whether cocaine altered the number of double-labeled PV cells surrounded by WFA (Table 1). While there were more PV/WFA double-labeled cells in the prelimbic region of the PFC compared with the infralimbic region, there were no differences in these PV/WFA double-labeled cells between saline- and cocaine-treated rats within either the prelimbic PFC or the infralimbic PFC.

### Cocaine exposure increases inhibitory and excitatory puncta on PV/WFA cells

**Figure 4.**
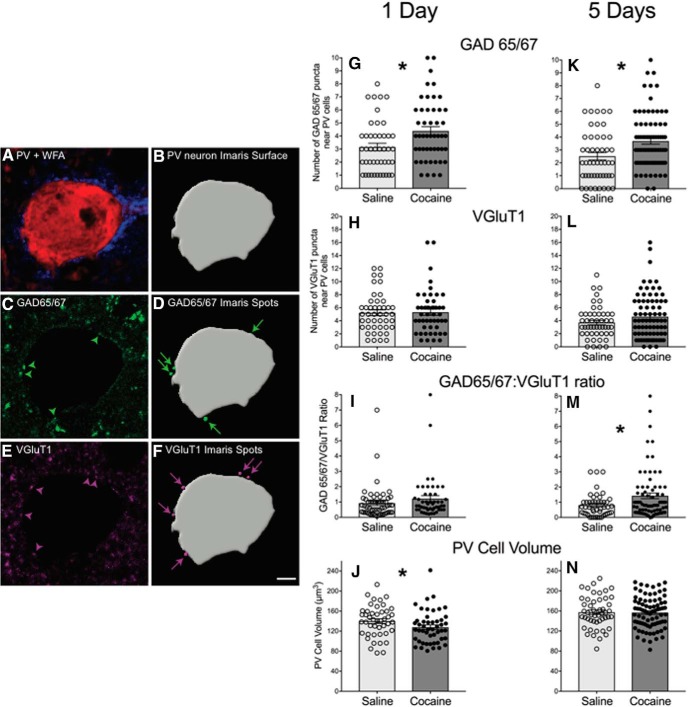
Cocaine alters intensity of GABAergic (GAD65/67) puncta near PV neurons surrounded by PNNs in the prelimbic PFC. Cells were visualized with confocal microscopy and analyzed using Imaris segmentation tools. ***A***, Representative PV neuron (red) surrounded by a PNN labeled with WFA (blue). ***B***, The PV neuron is traced and a surface is created. ***C***, The Mask Channel function was used to isolate GAD65/67 labeling to the area outside of the PV neuronal surface, and GAD65/67-labeled puncta (green arrowheads) were identified. ***D***, GAD65/67-labeled puncta (green arrowheads) were then thresholded using the Imaris Spots tool. Spots that met the size criteria and were located adjacent to the PV neuron surface were included in the analysis (green arrows). ***E***, ***F***, VGluT1-labeled puncta (magenta arrowheads) were segmented separately using the same protocol (***E***), and VGluT1 spots meeting our size and location criteria were included in the analysis (magenta arrows; ***F***). ***G–I***, One day of cocaine exposure increases GAD 65/67 puncta (saline, *N* = 3, 45 cells; cocaine, *N* = 3, 49 cells; ***G***), no change in VGluT1 puncta (***H***), and a trend toward an increase in the ratio of GAD65/67 to VGluT1 puncta (***I***). ***J***, One day of cocaine exposure decreases PV cell volume. ***K–M***, Five days of cocaine exposure increases GAD 65/67 puncta (saline, *N* = 4, 49 cells; cocaine, *N* = 6, 78 cells; ***K***), yields no change in VGluT1 puncta (***L***), and increases the ratio of GAD65/67 to VGluT1 puncta (***M***). ***N***, Five days of cocaine exposure does not alter PV cell volume. Data from bar graphs are the mean ± SEM. Scale bar, 3 µm. *p < 0.05 and #*p* < 0.1, compared with saline controls.

We focused all remaining studies on the prelimbic PFC because of the well described role of the prelimbic PFC projections to the nucleus accumbens in modulating cocaine-seeking behavior (McFarland and Kalivas, 2001; McLaughlin and See, 2003; McFarland et al., 2004; Ma et al., 2014), and our studies describing a key role for PNNs in the prelimbic but not the infralimbic PFC in cocaine-induced conditioned place preference (Slaker et al., 2015). To test whether cocaine exposure altered the type of input onto PV cells with PNNs within the prelimbic PFC, we measured GAD65/67-labeled inhibitory and VGluT1-labeled excitatory puncta near PV cells 2 h following 1 or 5 d of cocaine exposure. Figure 4*A–F* shows a representative example of a PV-labeled cell surrounded by a WFA-labeled PNN (Fig. 4*A*) that is apposed by GAD65/67 (Fig. 4*C*, green arrowheads) and VGluT1 (Fig. 4*E*, magenta arrowheads) puncta as well as how the analysis was conducted in Imaris (Fig. 4*B*,*D*,*F*). The number of GAD65/67 puncta apposed to PV cells increased after both 1 d (Fig. 4*G*; *p* = 0.0073) and 5 d (Fig. 4*K*; *p* = 0.0018) of cocaine exposure. The number of VGluT1 puncta did not change after 1 d of cocaine exposure (Fig. 4*H*) or after 5 d of cocaine exposure (Fig. 4*L*). There was a trend toward an increased ratio of GAD65/67/VGluT1 puncta near PV cells surrounded by WFA after 1 d of cocaine exposure (Fig. 4*I*; *p* = 0.0911), and this ratio was significantly increased after 5 d of cocaine exposure (Fig. 4*M*; *p* = 0.0407), suggesting that there was an increase in the inhibitory/excitatory ratio after repeated cocaine exposure. Inasmuch as these puncta represent new or impending synapse formation, our findings suggest that new inputs onto PV cells may occur rapidly (within 2 h). We also found an approximately 10% decrease in PV cell volume after 1 d of cocaine exposure (Fig. 4*J*; *p* = 0.0288), with no changes after 5 d of cocaine exposure.

### Electrophysiology

**Figure 5.**
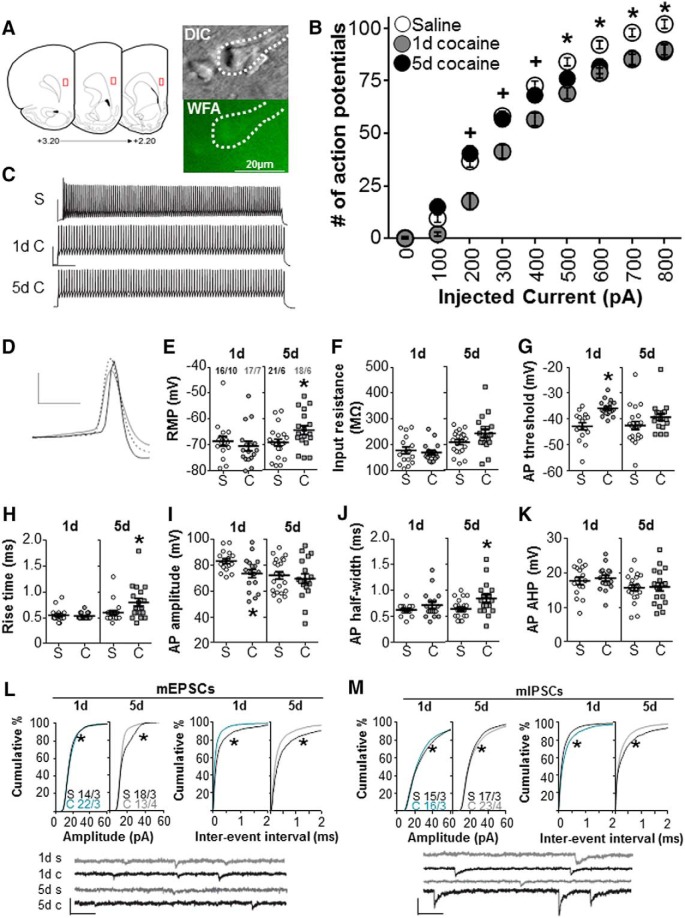
Intrinsic and synaptic properties of FSIs surrounded by PNNs exposed to cocaine in the prelimbic PFC. Data are the mean ± SEM. Fast-spiking interneuron properties 2 h following 1 or 5 d of saline or cocaine exposure. ***A***, Recording locations in layer V of the prelimbic PFC. ***B***, Action potentials recorded from FSIs surrounded by WFA. *p < 0.0001 comparing between saline and both cocaine groups; +*p* < 0.0001 comparing between saline and 1 d of cocaine exposure. ***C***, Trace examples of recorded FSIs at 800 pA. Calibration: 50 ms, 50 mV. ***D***, Trace example of first elicited action potential (AP). Dashed = saline exposure; gray = 1 d of cocaine exposure; black = 5 d of cocaine exposure. Calibration: 20 mV, 500 μs. ***E–K***, Intrinsic properties were as follows: resting membrane potential (***E***), input resistance (***F***), AP threshold (***G***), rise time (***H***), AP amplitude (***I***), AP half-width (***J***), and AP afterhyperpolarization potential (***K***), comparing acute and repeated cocaine (C) to acute and repeated saline controls (S), respectively. ***L***, Cumulative frequency plots of mEPSC amplitudes and interevent intervals. Trace examples are shown below. Calibration: 50 pA, 500 μs. ***M***, Cumulative frequency plots of mIPSC amplitudes and interevent intervals. Trace examples are shown below. The number of cells/number of rats used is shown (***E***, ***L***, ***M***).

We used whole-cell electrophysiology to determine whether an acute (1 d) or repeated (5 d) cocaine exposure influenced intrinsic excitability and/or synaptic transmission onto PNN-surrounded neurons in the deeper layers (V and VI) of the prelimbic PFC (Fig. 5*A*). The FSIs in this region of the cortex are highly likely to be PV-containing cells (Kawaguchi and Kubota, 1993). We recently found that >90% of FSIs recorded in these layers were surrounded by PNNs (unpublished observations). All recordings were made 2 h following acute (1 d) or repeated (5 d) cocaine exposure. Figure 5*B* shows that the number of current-induced action potentials was decreased after acute and repeated cocaine exposure compared with saline controls for WFA-labeled FSIs in the prelimbic PFC. There was a treatment effect (*F*_(2,61)_ = 7.72, *p* < 0.001), a time effect (*F*_(8,488)_ = 1150, *p* < 0.001), and a treatment × time interaction (*F*_(16 488)_ = 6.72, *p* < 0.001). Sidak’s *post hoc* analysis indicated that both acute and repeated cocaine exposure attenuated the number of action potentials elicited relative to saline controls. The decrease after acute cocaine exposure was observed after low levels of injected current, while the decrease after repeated cocaine exposure was observed only after higher levels of injected current (>500 pA). Figure 5*D* shows a trace example of the first elicited action potential from rats treated with either saline or acute or repeated cocaine exposure. Following acute cocaine exposure, the action potential amplitude was decreased (Fig. 5*I*; *p* = 0.0170); and following repeated cocaine exposure, the resting membrane potential was decreased (Fig. 5*E*; *p* = 0.0295), the half-width of action potentials was increased (Fig. 5*J*; *p* = 0.0170), and the rise time was increased (Fig. 5*H*; *p* = 0.0272). There were no differences between saline and cocaine groups for input resistance (Fig. 5*F*), action potential threshold (Fig. 5*G*), or action potential after hyperpolarization (Fig. 5*K*).

Additionally, we used miniature recordings to determine whether cocaine exposure influenced synaptic transmission onto PNN-surrounded FSIs. Pharmacological tools allowed us to isolate glutamatergic and GABAergic transmission. Figure 5*L* shows the mEPSC amplitude and frequency after cocaine treatment. We found that acute cocaine exposure produced a small but significant increase in mEPSC amplitude (*p* < 0.0001), but that repeated cocaine exposure decreased the mEPSC amplitude (*p* < 0.0001). The frequency of the mEPSC interevent interval was decreased after both acute (*p* < 0.0001) and repeated (*p* < 0.0001) cocaine exposure, suggesting greater excitatory input onto PV cells surrounded by WFA. Figure 5*M* shows the mIPSC amplitude and frequency after cocaine treatment. Acute cocaine treatment decreased the mIPSC amplitude (*p* < 0.0001), but, in contrast, repeated cocaine exposure increased the mIPSC amplitude (*p* < 0.0001). The frequency of the mIPSC interevent interval was increased after acute cocaine exposure (*p* < 0.0001) but was decreased after repeated cocaine exposure (*p* = 0.0015). Overall, these findings indicate that, while repeated cocaine exposure may increase both excitatory and inhibitory input onto FSIs, the postsynaptic excitatory response is reduced, and the combination of both an increase in inhibitory input and an increase in mIPSC amplitude would be expected to inhibit the output of FSIs.

### Composition of glycosaminoglycans, hyaluronic acid, and chondroitin sulfates following a single or repeated cocaine exposure

PNNs are composed of interacting GAGs. HA is produced in the membrane and extruded to produce an ECM backbone to which CSPG and HS proteoglycan are bound and stabilized through link proteins. The position of the sulfate groups on CSPGs changes patterns during development (Carulli et al., 2010), with the 0S and 6S positions prevalent during periods of high plasticity and the 4S position being dominant during periods of low plasticity (Carulli et al., 2010). Here we determined the impact of 1 or 5 d of saline or cocaine treatment on levels of GAGs, HS, and CSs 2 h later. We used mPFC brain punches, which comprised primarily the prelimbic PFC but also some portions of the infralimbic PFC. Table 2 shows that approximately half of the total GAG composition was in the form of CS, with the remaining half being both HS and HA. This population did not shift 2 h after acute or repeated cocaine exposure. We also assessed the sulfation patterns of CSs and HSs. For CSs, the 4S position was the most prevalent sulfation position (90%). However, there was no difference between treatment groups for sulfate position on CSs. Additionally, on HSs, the 0S position was the most prevalent sulfation position, with >60% of all sulfates at this position. There was no difference between treatment groups for sulfate position on HSs (Table 2). Thus, the changes in PNNs found after cocaine exposure were not reflected in measurements that included both PNNs and the loose ECM.

**Table 2.** Composition of GAGs in mPFC after 1 or 5 d of saline or cocaine

		1 d		5 d	
	Group	Saline	Cocaine	Saline	Cocaine
Total GAG composition	HS	17.9 ± 4.1%	19.3 ± 6.9%	18.4 ± 2.2%	17.2 ± 2.7%
	CS	54.3 ± 8.2	51.7 ± 11.6	52.8 ± 4.1	56.8 ± 4.4
	HA	27.8 ± 10.6	29.0 ± 13.6	28.8 ± 6.2	26.0 ± 6.9
HS composition	TriS	2.3 ± 1.2%	2.2 ± 1.6%	1.6 ± 0.4%	1.8 ± 0.2%
	NS6S	4.4 ± 1.3%	3.9 ± 1.8	3.9 ± 0.7	4.2 ± 0.6
	NS2S	11.9 ± 3.5%	10.8 ± 4.1	8.7 ± 1.2	10.5 ± 0.8
	NS	17.8 ± 2.9	16.7 ± 4.8	17.7 ± 1.3	18.2 ± 1.1
	2S6S	0.1 ± 0.1	0.2 ± 0.1	0.1 ± 0.1	0.1 ± 0.1
	6S	2.7 ± 1.2	2.6 ± 1.3	2.8 ± 0.5	3.0 ± 0.3
	2S	0.5 ± 0.1	0.3 ± 0.2	0.5 ± 0.1	0.4 ± 0.1
	0S	60.4 ± 8.9	63.2 ± 12.4	64.6 ± 3.6	61.7 ± 2.7
CS composition	2S4S	0.2 ± 0.1%	0.2 ± 0.1%	0.2 ± 0.1%	0.3 ± 0.1%
	2S6S	1.1 ± 0.5	1.0 ± 0.5	0.8 ± 0.3	1.0 ± 0.3
	4S6S	1.1 ± 0.2	1.1 ± 0.3	0.9 ± 0.1	1.0 ± 0.1
	4S	89.8 ± 3.9	89.5 ± 3.4	90.3 ± 1.5	89.1 ± 2.0
	6S	2.4 ± 0.9	1.9 ± 0.8	2.2 ± 0.5	2.7 ± 0.9
	2S	0.2 ± 0.1	0.1 ± 0.1	0.2 ± 0.1	0.1 ± 0.1
	0S	5.2 ± 2.5	6.1 ± 3.7	5.3 ± 1.5	5.8 ± 0.9

## Discussion

Recent studies have shown that other drugs of abuse (cocaine, heroin, nicotine, and alcohol) alter the intensity of PNNs and/or their components, with the direction of the changes dependent on the drug, the extent of exposure and withdrawal, and the brain region (Van den Oever et al., 2010b; Coleman et al., 2014; Vazquez-Sanroman et al., 2015b, 2017; Carbo-Gas et al., 2017). However, the functional state of PV FSIs surrounded by PNNs has not been investigated. Here we provide evidence that the intensity of PNN and PV labeling responds to cocaine exposure with temporal specificity in the prelimbic PFC and infralimbic PFC. Changes in the intensity of PNNs and PV occurred within 2 h of a single cocaine exposure and generally paralleled each other, with PV intensity changes occurring several hours after PNN changes. Acute cocaine exposure decreased PNN and PV intensity, while repeated cocaine exposure increased PNN and PV intensity. In the prelimbic PFC, acute cocaine decreased the excitability of FSIs surrounded by PNNs, but, inconsistent with this decreased excitability, produced synaptic changes indicative of increased glutamate release and decreased GABA release from inputs to these cells. This disconnect between quantal input to the cell and the overall excitability could be due to changes in the efficacy of ionic influx after quantal events, perhaps due to changes in intracellular levels of PV, which functions as a calcium buffer (Caillard et al., 2000). However, repeated cocaine exposure produced changes consistent with decreases in PV/PNN cell excitability after cocaine exposure, including a decrease in evoked action potentials of FSIs, an increase in the ratio of GAD65/67 to VGluT1 puncta, an increase in both the frequency and amplitude of mIPSCs, and a reduction in the amplitude of mEPSCs.

### Cocaine-induced changes in PNN and PV intensity

Previous work reported a positive correlation between the intensity of PV and PNNs (Yamada et al., 2015), in accordance with our current findings. Cocaine-induced changes in PV intensity generally followed those in PNNs, suggesting a potential codependence of PV and PNNs. PNNs are believed to protect underlying PV neurons from oxidative stress (Cabungcal et al., 2013b), and a higher firing frequency in PV FSIs may require higher PV content to bind intracellular calcium and promote activity-dependent production of PNNs (Dityatev et al., 2007) that sequester calcium (Brückner et al., 1993). Recent work demonstrated that experience alters the activity of PV cells in the hippocampus through the PNN component brevican (Favuzzi et al., 2017), which mediates changes through altered AMPA receptors and voltage-gated potassium channels, which are critical for the fast spiking of PV cells. While we did not measure individual CSPGs within PNNs, we assessed total GAG composition in the total ECM (which includes PNNs) from tissue punches containing the prelimbic PFC. No changes in these components were found after acute or repeated cocaine exposure, suggesting that any changes in PNN intensity are likely specific to these structures rather than to the loose ECM.

In some instances, we found a correlation between the intensity of WFA or PV staining and cocaine-induced locomotor behavior in the prelimbic and infralimbic PFC (Fig. 1-1, available at https://doi.org/10.1523/ENEURO.0221-18.2018.f1-1). The strongest correlations and most consistently observed changes appeared to be in WFA^+^/PV^+^ cells in the prelimbic PFC just 2 h after 5 d of cocaine exposure. WFA staining around PV cells was strongly correlated with days 2 and 3 of cocaine exposure, and nearly correlated with day 5 of cocaine-induced locomotor activity, suggesting that locomotor activity after cocaine injection on days 2 and 3 may reflect cocaine-induced plasticity within PNNs that in turn shapes prelimbic PFC output to modulate locomotor activity.

### Cocaine-induced changes in intrinsic and synaptic properties of FSIs

The findings here expand on those from out our previous study in which we found that repeated cocaine exposure reduced the mIPSC frequency onto pyramidal neurons in the prelimbic PFC (Slaker et al., 2015). This attenuation may be due to a cocaine-induced reduction in the firing of PNN-surrounded PV neurons mediated by increased GABA release and increased postsynaptic responsiveness to GABA on these PV neurons, along with an increase in the ratio of GAD65/67 to VGluT1 puncta. Of note, our analysis accounted for puncta only near the cell body, which may not reflect changes in punta around dendrites that contribute to firing.

The electrophysiological findings in FSIs, which are likely PV-containing cells (Kawaguchi and Kubota, 1993), are consistent with the idea that an increase in the inhibitory/excitatory balance in PV/PNN cells reduces inhibitory output to pyramidal neurons, contributing to the hyperexcitability of these neurons that we and others have reported (Dong et al., 2005; Nasif et al., 2005; Huang et al., 2007; Hearing et al., 2013; Slaker et al., 2015) following repeated, noncontingent cocaine exposure. These studies are also consistent with the finding that targeted optogenetic stimulation of PV FSIs globally inhibits prelimbic PFC network activity (Sparta et al., 2014). However, previous work showed that a cocaine challenge elevates extracellular GABA levels in the mPFC (Jayaram and Steketee, 2005) in cocaine-sensitized rats, although the contribution by FSIs to the elevated extracellular GABA levels could not be discerned in that study. FSIs in particular may be modulated by cocaine to quickly shut off the activity of pyramidal neurons (Lapish et al., 2007), and repeated cocaine treatment may reduce this ability to inhibit pyramidal neurons.

The changes in electrophysiological properties of FSIs 2 h after repeated cocaine exposure suggest that cocaine alters FSIs differently from paradigms that examine changes after withdrawal from cocaine. Campanac and Hoffman (2013) found that 5 d of cocaine injections followed by 10–13 d of withdrawal increased the spike number and attenuated mEPSC amplitude and frequency. We observed a decrease in spike number with a decrease in mEPSC amplitude and an increase in the mEPSC frequency after 5 d of cocaine exposure. Although these studies did not use WFA to label PNNs, a vast majority of these cells likely would have stained positive for WFA because >90% of WFA-labeled neurons are FSIs within layer V of the prelimbic PFC (unpublished observations). Together, these findings suggest that both cocaine exposure and subsequent withdrawal influence the intrinsic firing and synaptic transmission of FSIs, but that withdrawal periods facilitate additional plasticity. Nonetheless, the events occurring before withdrawal may give clues to the sequence of early events involved in disrupted PV cell-associated circuits contributing to aberrant learning processes after cocaine exposure.

### Mechanistic considerations

Previous studies have shown that PNNs or their components influence intrinsic firing and synaptic transmission (Dityatev et al., 2007; Van den Oever et al., 2010a; Slaker et al., 2015; Balmer, 2016; Carstens et al., 2016; Favuzzi et al., 2017). Full degradation of PNNs has been shown to both decrease (Balmer, 2016) and increase (Dityatev et al., 2007) the firing of cultured neurons and to impair the induction of long-term potentiation (Carstens et al., 2016), with the difference likely dependent on the brain region and time interval after PNN degradation. Our data show that cocaine exposure in the intact brain (no exogenous enzymatic PNN degradation) attenuates the firing of PNN-surrounded FSIs in the prelimbic PFC. Thus, cocaine-induced changes in the intensity of PNNs may influence firing, which is consistent with the above studies in which PNNs are completely degraded and with a recent report (Favuzzi et al., 2017) demonstrating a role for the PNN component brevican in regulating firing in PV cells and excitatory input to PV cells, in turn altering the intrinsic properties of PV neurons, possibly via K_v_1.1 or K_v_3.1b channels. Not readily explainable are our observations that repeated cocaine exposure decreased the firing of and increased the inhibitory input to FSIs in the prelimbic PFC at the same time point that we found increased PV and PNN intensity. These findings suggest that transient changes in PNN or PV intensity may not be coordinated temporally with the changes in electrophysiological measures or that the increases in intensity are compensatory responses to cocaine exposure.

A key mechanism that may link cocaine exposure to changes in PNN and/or PV intensity is cocaine-induced oxidative stress (Dietrich et al., 2005; Jang et al., 2015). A central role for PNNs is their protection of underlying neurons from oxidative stress (Morawski et al., 2004; Cabungcal et al., 2013b; Suttkus et al., 2014). PNNs, however, are themselves susceptible to oxidative stress (Cabungcal et al., 2013b). Thus, while the impact of cocaine on PNNs is transient and may occur in response to oxidative stress, these responses may set the stage for a cascade of downstream consequences, including partial degradation of PNNs that allows for new synaptic inputs onto PV cells after acute cocaine exposure. The acute cocaine-induced decrease in PV cell volume in the prelimbic PFC may reflect a cocaine-induced oxidative stress effect. In contrast, the increase in PNN intensity after repeated cocaine exposure, although relatively small, may restrict new synaptic inputs by the binding of inhibitory molecules such as Sema3A, by physical constraints (Wang and Fawcett, 2012; Vo et al., 2013), and/or by restricting the lateral movement of AMPA receptors important for short-term plasticity (Frischknecht et al., 2009). Changes in PV cell volume do not appear to underlie changes in PNN intensity or in the intrinsic or synaptic properties after repeated cocaine exposure, since PV cell volumes were not different between saline controls and cocaine-treated rats after repeated cocaine exposure.

### Conclusions

Here we demonstrated that the intensity of PNNs and PV rapidly decreases in the prelimbic and infralimbic PFC following a novel cocaine exposure but increases following repeated cocaine exposure. The decreased PNN intensity in response to a single cocaine injection may promote early changes that enable new synaptic input, recapitulating early development and in accordance with the rejuvenation hypothesis of cocaine addiction (Dong and Nestler, 2014). In the prelimbic PFC, repeated cocaine exposure increases inhibitory input to PV cells surrounded by PNNs, which in turn may promote the hyperexcitability of pyramidal neurons that we and others previously reported and that promote reinstatement to drug-seeking behavior. While some of these changes may be transient, the rapid responses to cocaine may alter the network stability of PV FSIs that partially set into motion the persistent and chronic nature of drug addiction. Importantly, PV FSIs drive gamma oscillations that promote cognitive processing (Womelsdorf et al., 2007; Sohal et al., 2009), and adaptations in these PV FSIs may also underlie the cognitive impairment in individuals addicted to cocaine (Potvin et al., 2014).

## Synthesis

Reviewing Editor: Christian Lüscher, University of Geneva Decisions are customarily a result of the Reviewing Editor and the peer reviewers coming together and discussing their recommendations until a consensus is reached. When revisions are invited, a fact-based synthesis statement explaining their decision and outlining what is needed to prepare a revision will be listed below. The following reviewer(s) agreed to reveal their identity: Erin Calipari, Jeffery Steketee. Note: If this manuscript was transferred from JNeurosci and a decision was made to accept the manuscript without peer review, a brief statement to this effect will instead be what is listed below.

Please consider the following suggestions:

- Was motor activity data collected from all animals used in this report, or just a subset? I ask this question since experiments were conducted at different universities. If it was a subset, the authors should indicate which studies included motor activity monitoring.

- In the results section, the authors should describe how one interprets figures 2 and 3. Does significance refer to where the peaks are relative to each other, the amount of space under each curve or some other measure?

- The following two sentences appear to be making contradictory statements. Please clarify. “We consistently found more PV cells double-labeled with WFA in the prelimbic compared to the infralimbic PFC, in accordance with previous studies examining PNNs in the mPFC (Slaker et al., 2015). There were no differences in PV/WFA double-labeled cell numbers in either the prelimbic or infralimbic PFC.”

- On page 14, 5 lines from the bottom, the authors state that the effects of cocaine may be sustained for at least 5 days. Based on the experimental design, I do not believe the authors can make this claim.

- In figure 1 it would be helpful to provide data showing the relationship between the immunohistochemistry and behavior. For example, is PV or WFA intensity correlated with sensitization? If the authors have the data, this should be done for all of the behavioral time points, and for the data on the last day it could be done for both the locomotor activity induced on the last day or the change in motor effects over time. This would greatly strengthen the conclusions within the manuscript.

- What are the PV intensity measurements reflective of - changes in morphology of these neurons (i.e. neuron size), or just changes in PV levels within the same neurons? The authors suggest that higher firing frequency may increase PV content, which is interesting, but can the authors be certain that the morphology or size of these neurons has not changed and is influencing these effects?

- One concern is the overstatement of the data from figure 4: The authors state that cocaine alters intensity of GABAergic (GAD65/67) puncta onto PV neurons. While it is clear that there are differences in puncta with the resolution of a confocal you cannot be certain that this is actually synapsing onto PV neurons with this method. This wording should be softened.

## Author Response

We thank the reviewers for their insights and suggestions that have now improved the clarity and enhanced the manuscript. We address the comments individually below and now include the following revised Figures: 1) A new graph added to Figure 1 (Figure 1D), which shows the correlation between WFA/PV staining intensity and cocaine-induced locomotor activity); 2) a new Extended Data Figure 1-1, which is the table reporting all significant correlations between staining intensity and cocaine-induced locomotor activity for all treatments, time points, and the two brain regions; 3) new bar graphs added as insets to each graph shown in Figures 2 and 3 to aid in the visualization of increases/decreases in WFA and PV intensity; and 4) two new graphs added to Figure 4 (Figures 4J and N), which show PV cell volume in the same groups analyzed for inhibitory and excitatory puncta. All changes within the manuscript are indicated in blue font.

1. Was motor activity data collected from all animals used in this report, or just a subset? I ask this question since experiments were conducted at different universities. If it was a subset, the authors should indicate which studies included motor activity monitoring.

The motor activity was collected from all animals used in these studies (not a subset). We now state in the Results section (page 12): “We show the locomotor activity of every rat used in these studies after acute and repeated saline or cocaine, the latter of which produces behavioral sensitization (Robinson and Berridge, 1993).”

2. In the results section, the authors should describe how one interprets figures 2 and does significance refer to where the peaks are relative to each other, the amount of space under each curve or some other measure?

The significance (and asterisk on Figures 2 and 3) refer to a change in the distribution of cells with particular intensities binned into groups on the x-axis. The asterisk indicates a difference between saline- and cocaine-treated rats. However, to aid in visualization of these distributions (whether there is overall a decrease or increase in intensity) we have now added bar graphs as insets to each graph.

3. The following two sentences appear to be making contradictory statements. Please clarify. “We consistently found more PV cells double-labeled with WFA in the prelimbic compared to the infralimbic PFC, in accordance with previous studies examining PNNs in the mPFC (Slaker et al., 2015). There were no differences in PV/WFA double-labeled cell numbers in either the prelimbic or infralimbic PFC.”

We regret the confusion, and have now clarified that, “while there were more PV/WFA double-labeled cells in the prelimbic region of the PFC compared with the infralimbic region, there were no differences in these PV/WFA double-labeled cells between saline and cocaine-treated rats within either the prelimbic PFC or the infralimbic PFC.” (Please see Results section, page 15).

4. On page 14, 5 lines from the bottom, the authors state that the effects of cocaine may be sustained for at least 5 days. Based on the experimental design, I do not believe the authors can make this claim.

We have now omitted this part of the sentence.

5. In figure 1 it would be helpful to provide data showing the relationship between the immunohistochemistry and behavior. For example, is PV or WFA intensity correlated with sensitization? If the authors have the data, this should be done for all of the behavioral time points, and for the data on the last day it could be done for both the locomotor activity induced on the last day or the change in motor effects over time. This would greatly strengthen the conclusions within the manuscript.

We thank the reviewer for the suggestion. We have now generated correlations for all combinations of intensity analysis and locomotor activity (intensity of single- and double-labeled WFA cells and single- and double-labeled PV cells in the prelimbic PFC and infralimbic PFC at 2 hr and 24 hr after 1 day and 5 days of cocaine, including day 5 -- day D1 differences). We ended up with 12 significant correlations, which are now reported in the Extended Date (Figure 1-1) and have chosen to show the strongest correlation found, which was between WFA/PV double-labeled cell intensity and Day 2 of cocaine-induced locomotor activity 2 h after 5 days of cocaine exposure. This particular correlation is shown in Figure 1D. We also report the findings in the Results section (see pages 12-13) and in the Discussion (see page 19). Unfortunately, since we did not sample WFA/PV staining after each day of cocaine injection, it is somewhat speculative to draw a conclusion from these findings, but we state in the Discussion on p. 19 the following: “In some instances, we found a correlation between the intensity of WFA or PV staining and cocaine-induced locomotor behavior in the prelimbic and infralimbic PFC (Figure 1-1). The strongest correlations and most consistently observed changes appeared to be in WFA+/PV+ cells in the prelimbic PFC just 2 h after 5 days of cocaine exposure. WFA staining around PV cells was strongly correlated with Days 2 and 3, and nearly correlated with Day 5 of cocaine-induced locomotor activity, suggesting that locomotor activity after cocaine injection on Days 2 and 3 may reflect cocaine-induced plasticity within PNNs that in turn shape prelimbic PFC output to modulate locomotor activity.”

6. What are the PV intensity measurements reflective of - changes in morphology of these neurons (i.e. neuron size), or just changes in PV levels within the same neurons? The authors suggest that higher firing frequency may increase PV content, which is interesting, but can the authors be certain that the morphology or size of these neurons has not changed and is influencing these effects?

This is an interesting question, and we have data showing that the PV cell volume, which was measured in cells in which puncta analysis was conducted, was reduced by about 10% after acute cocaine but was not changed after repeated cocaine. This information has now been added to the Results (page 15), Discussion (p 21), and is also presented as new graphs in Figure 4 (Figures 4J and N).

7. One concern is the overstatement of the data from figure 4: The authors state that cocaine alters intensity of GABAergic (GAD65/67) puncta onto PV neurons. While it is clear that there are differences in puncta with the resolution of a confocal you cannot be certain that this is actually synapsing onto PV neurons with this method. This wording should be softened.

We regret the overstatement; this has now been changed to “puncta near PV cells” throughout the manuscript and in the Figure legend for Figure 4.

## References

[B1] Balmer TS (2016) Perineuronal nets enhance the excitability of fast-spiking neurons. eNeuro 3:ENEURO.0112-16.2016. 10.1523/ENEURO.0112-16.2016 PMC4987413 27570824

[B2] Balmer TS, Carels VM, Frisch JL, Nick TA (2009) Modulation of perineuronal nets and parvalbumin with developmental song learning. J Neurosci 29:12878–12885. 10.1523/JNEUROSCI.2974-09.2009 19828802 PMC2769505

[B3] Banerjee SB, Gutzeit VA, Baman J, Aoued HS, Doshi NK, Liu RC, Ressler KJ (2017) Perineuronal nets in the adult sensory cortex are necessary for fear learning. Neuron 95:169–179.e3. 10.1016/j.neuron.2017.06.007 28648500 PMC5548423

[B4] Brückner G, Brauer K, Härtig W, Wolff JR, Rickmann MJ, Derouiche A, Delpech B, Girard N, Oertel WH, Reichenbach A (1993) Perineuronal nets provide a polyanionic, glia-associated form of microenvironment around certain neurons in many parts of the rat brain. Glia 8:183–200. 10.1002/glia.440080306 7693589

[B5] Cabungcal JH, Steullet P, Kraftsik R, Cuenod M, Do KQ (2013a) Early-life insults impair parvalbumin interneurons via oxidative stress: reversal by N-acetylcysteine. Biol Psychiatry 73:574–582. 10.1016/j.biopsych.2012.09.020 23140664

[B6] Cabungcal JH, Steullet P, Morishita H, Kraftsik R, Cuenod M, Hensch TK, Do KQ (2013b) Perineuronal nets protect fast-spiking interneurons against oxidative stress. Proc Natl Acad Sci U S A 110:9130–9135. 10.1073/pnas.1300454110 23671099 PMC3670388

[B7] Caillard O, Moreno H, Schwaller B, Llano I, Celio MR, Marty A (2000) Role of the calcium-binding protein parvalbumin in short-term synaptic plasticity. Proc Natl Acad Sci U S A 97:13372–13377. 10.1073/pnas.230362997 11069288 PMC27231

[B8] Campanac E, Hoffman DA (2013) Repeated cocaine exposure increases fast-spiking interneuron excitability in the rat medial prefrontal cortex. J Neurophysiol 109:2781–2792. 10.1152/jn.00596.2012 23486201 PMC3680802

[B9] Carbo-Gas M, Moreno-Rius J, Guarque-Chabrera J, Vazquez-Sanroman D, Gil-Miravet I, Carulli D, Hoebeek F, De Zeeuw C, Sanchis-Segura C, Miquel M (2017) Cerebellar perineuronal nets in cocaine-induced pavlovian memory: site matters. Neuropharmacology 125:166–180. 10.1016/j.neuropharm.2017.07.009 28712684

[B10] Carstens KE, Phillips ML, Pozzo-Miller L, Weinberg RJ, Dudek SM (2016) Perineuronal nets suppress plasticity of excitatory synapses on CA2 pyramidal neurons. J Neurosci 36:6312–6320. 10.1523/JNEUROSCI.0245-16.2016 27277807 PMC4899529

[B11] Carulli D, Pizzorusso T, Kwok JC, Putignano E, Poli A, Forostyak S, Andrews MR, Deepa SS, Glant TT, Fawcett JW (2010) Animals lacking link protein have attenuated perineuronal nets and persistent plasticity. Brain 133:2331–2347. 10.1093/brain/awq145 20566484

[B12] Chen H, He D, Lasek AW (2015) Repeated binge drinking increases perineuronal nets in the insular cortex. Alcohol Clin Exp Res 39:1930–1938. 10.1111/acer.12847 26332441 PMC4592458

[B13] Coleman LG Jr, Liu W, Oguz I, Styner M, Crews FT (2014) Adolescent binge ethanol treatment alters adult brain regional volumes, cortical extracellular matrix protein and behavioral flexibility. Pharmacol Biochem Behav 116:142–151. 10.1016/j.pbb.2013.11.021 24275185 PMC3913047

[B14] Dietrich JB, Mangeol A, Revel MO, Burgun C, Aunis D, Zwiller J (2005) Acute or repeated cocaine administration generates reactive oxygen species and induces antioxidant enzyme activity in dopaminergic rat brain structures. Neuropharmacology 48:965–974. 10.1016/j.neuropharm.2005.01.018 15857623

[B15] Dityatev A, Brückner G, Dityateva G, Grosche J, Kleene R, Schachner M (2007) Activity-dependent formation and functions of chondroitin sulfate-rich extracellular matrix of perineuronal nets. Dev Neurobiol 67:570–588. 10.1002/dneu.20361 17443809

[B16] Donato F, Rompani SB, Caroni P (2013) Parvalbumin-expressing basket-cell network plasticity induced by experience regulates adult learning. Nature 504:272–276. 10.1038/nature12866 24336286

[B17] Dong Y, Nestler EJ (2014) The neural rejuvenation hypothesis of cocaine addiction. Trends Pharmacol Sci 35:374–383. 10.1016/j.tips.2014.05.005 24958329 PMC4119850

[B18] Dong Y, Nasif FJ, Tsui JJ, Ju WY, Cooper DC, Hu XT, Malenka RC, White FJ (2005) Cocaine-induced plasticity of intrinsic membrane properties in prefrontal cortex pyramidal neurons: adaptations in potassium currents. J Neurosci 25:936–940. 10.1523/JNEUROSCI.4715-04.2005 15673674 PMC6725625

[B19] Favuzzi E, Marques-Smith A, Deogracias R, Winterflood CM, Sánchez-Aguilera A, Mantoan L, Maeso P, Fernandes C, Ewers H, Rico B (2017) Activity-dependent gating of parvalbumin interneuron function by the perineuronal net protein brevican. Neuron 95:639–655.e10. 10.1016/j.neuron.2017.06.028 28712654

[B20] Fawcett J (2009) Molecular control of brain plasticity and repair. Prog Brain Res 175:501–509. 10.1016/S0079-6123(09)17534-9 19660677

[B21] Foscarin S, Ponchione D, Pajaj E, Leto K, Gawlak M, Wilczynski GM, Rossi F, Carulli D (2011) Experience-dependent plasticity and modulation of growth regulatory molecules at central synapses. PLoS One 6:e16666. 10.1371/journal.pone.0016666 21304956 PMC3031615

[B22] Foscarin S, Raha-Chowdhury R, Fawcett JW, Kwok JCF (2017) Brain ageing changes proteoglycan sulfation, rendering perineuronal nets more inhibitory. Aging (Albany NY) 9:1607–1622. 10.18632/aging.101256 28657900 PMC5509459

[B23] Frischknecht R, Heine M, Perrais D, Seidenbecher CI, Choquet D, Gundelfinger ED (2009) Brain extracellular matrix affects AMPA receptor lateral mobility and short-term synaptic plasticity. Nat Neurosci 12:897–904. 10.1038/nn.2338 19483686

[B24] Gogolla N, Caroni P, Lüthi A, Herry C (2009) Perineuronal nets protect fear memories from erasure. Science 325:1258–1261. 10.1126/science.1174146 19729657

[B25] Härtig W, Brauer K, Brückner G (1992) Wisteria floribunda agglutinin-labelled nets surround parvalbumin-containing neurons. Neuroreport 3:869–872. 1421090 10.1097/00001756-199210000-00012

[B26] Hearing M, Kotecki L, Marron Fernandez de Velasco E, Fajardo-Serrano A, Chung HJ, Luján R, Wickman K (2013) Repeated cocaine weakens GABA(B)-Girk signaling in layer 5/6 pyramidal neurons in the prelimbic cortex. Neuron 80:159–170. 10.1016/j.neuron.2013.07.019 24094109 PMC3793643

[B27] Hegarty DM, Tonsfeldt K, Hermes SM, Helfand H, Aicher SA (2010) Differential localization of vesicular glutamate transporters and peptides in corneal afferents to trigeminal nucleus caudalis. J Comp Neurol 518:3557–3569. 10.1002/cne.22414 20593358 PMC2933108

[B28] Hegarty DM, Hermes SM, Largent-Milnes TM, Aicher SA (2014) Capsaicin-responsive corneal afferents do not contain TRPV1 at their central terminals in trigeminal nucleus caudalis in rats. J Chem Neuroanat 61-62:1–12. 10.1016/j.jchemneu.2014.06.006 24996127 PMC4268050

[B29] Huang CC, Lin HJ, Hsu KS (2007) Repeated cocaine administration promotes long-term potentiation induction in rat medial prefrontal cortex. Cereb Cortex 17:1877–1888. 10.1093/cercor/bhl096 17050645

[B30] Jang EY, Ryu YH, Lee BH, Chang SC, Yeo MJ, Kim SH, Folsom RJ, Schilaty ND, Kim KJ, Yang CH, Steffensen SC, Kim HY (2015) Involvement of reactive oxygen species in cocaine-taking behaviors in rats. Addict Biol 20:663–675. 10.1111/adb.12159 24975938 PMC4843775

[B31] Jayaram P, Steketee JD (2005) Effects of cocaine-induced behavioural sensitization on GABA transmission within rat medial prefrontal cortex. Eur J Neurosci 21:2035–2039. 10.1111/j.1460-9568.2005.04000.x 15869498

[B32] Kawaguchi Y, Kubota Y (1993) Correlation of physiological subgroupings of nonpyramidal cells with parvalbumin- and calbindinD28k-immunoreactive neurons in layer V of rat frontal cortex. J Neurophysiol 70:387–396. 10.1152/jn.1993.70.1.387 8395585

[B33] Kroener S, Lavin A (2010) Altered dopamine modulation of inhibition in the prefrontal cortex of cocaine-sensitized rats. Neuropsychopharmacology 35:2292–2304. 10.1038/npp.2010.107 20664581 PMC2939941

[B34] Kwok JC, Dick G, Wang D, Fawcett JW (2011) Extracellular matrix and perineuronal nets in CNS repair. Dev Neurobiol 71:1073–1089. 10.1002/dneu.20974 21898855

[B35] Lapish CC, Kroener S, Durstewitz D, Lavin A, Seamans JK (2007) The ability of the mesocortical dopamine system to operate in distinct temporal modes. Psychopharmacology 191:609–625. 10.1007/s00213-006-0527-8 17086392 PMC5509053

[B36] Lee AT, Gee SM, Vogt D, Patel T, Rubenstein JL, Sohal VS (2014) Pyramidal neurons in prefrontal cortex receive subtype-specific forms of excitation and inhibition. Neuron 81:61–68. 10.1016/j.neuron.2013.10.031 24361076 PMC3947199

[B37] Le Moine C, Gaspar P (1998) Subpopulations of cortical GABAergic interneurons differ by their expression of D1 and D2 dopamine receptor subtypes. Mol Brain Res 58:231–236. 10.1016/S0169-328X(98)00118-1 9685656

[B38] Ma YY, Lee BR, Wang X, Guo C, Liu L, Cui R, Lan Y, Balcita-Pedicino JJ, Wolf ME, Sesack SR, Shaham Y, Schlüter OM, Huang YH, Dong Y (2014) Bidirectional modulation of incubation of cocaine craving by silent synapse-based remodeling of prefrontal cortex to accumbens projections. Neuron 83:1453–1467. 10.1016/j.neuron.2014.08.023 25199705 PMC4295617

[B39] Madinier A, Quattromani MJ, Sjölund C, Ruscher K, Wieloch T (2014) Enriched housing enhances recovery of limb placement ability and reduces aggrecan-containing perineuronal nets in the rat somatosensory cortex after experimental stroke. PLoS One 9:e93121. 10.1371/journal.pone.0093121 24664200 PMC3963994

[B40] McFarland K, Kalivas PW (2001) The circuitry mediating cocaine-induced reinstatement of drug-seeking behavior. J Neurosci 21:8655–8663. 11606653 10.1523/JNEUROSCI.21-21-08655.2001 PMC6762812

[B41] McFarland K, Davidge SB, Lapish CC, Kalivas PW (2004) Limbic and motor circuitry underlying footshock-induced reinstatement of cocaine-seeking behavior. J Neurosci 24:1551–1560. 10.1523/JNEUROSCI.4177-03.2004 14973230 PMC6730472

[B42] McLaughlin J, See RE (2003) Selective inactivation of the dorsomedial prefrontal cortex and the basolateral amygdala attenuates conditioned-cued reinstatement of extinguished cocaine-seeking behavior in rats. Psychopharmacology 168:57–65. 10.1007/s00213-002-1196-x 12845418

[B43] Morawski M, Brückner MK, Riederer P, Brückner G, Arendt T (2004) Perineuronal nets potentially protect against oxidative stress. Exp Neurol 188:309–315. 10.1016/j.expneurol.2004.04.017 15246831

[B44] Nasif FJ, Sidiropoulou K, Hu XT, White FJ (2005) Repeated cocaine administration increases membrane excitability of pyramidal neurons in the rat medial prefrontal cortex. J Pharmacol Exp Ther 312:1305–1313. 10.1124/jpet.104.075184 15574686

[B45] Paxinos GWC (1998) The rat brain in stereotaxic coordinates, Ed 4 New York, NY: Academic.

[B46] Pizzorusso T, Medini P, Berardi N, Chierzi S, Fawcett JW, Maffei L (2002) Reactivation of ocular dominance plasticity in the adult visual cortex. Science 298:1248–1251. 10.1126/science.1072699 12424383

[B47] Potvin S, Stavro K, Rizkallah E, Pelletier J (2014) Cocaine and cognition: a systematic quantitative review. J Addict Med 8:368–376. 10.1097/ADM.0000000000000066 25187977

[B48] Robinson TE, Berridge KC (1993) The neural basis of drug craving: an incentive-sensitization theory of addiction. Brain Res Brain Res Rev 18:247–291. 8401595 10.1016/0165-0173(93)90013-p

[B49] Romberg C, Yang S, Melani R, Andrews MR, Horner AE, Spillantini MG, Bussey TJ, Fawcett JW, Pizzorusso T, Saksida LM (2013) Depletion of perineuronal nets enhances recognition memory and long-term depression in the perirhinal cortex. J Neurosci 33:7057–7065. 10.1523/JNEUROSCI.6267-11.2013 23595763 PMC3655688

[B50] Slaker M, Churchill L, Todd RP, Blacktop JM, Zuloaga DG, Raber J, Darling RA, Brown TE, Sorg BA (2015) Removal of perineuronal nets in the medial prefrontal cortex impairs the acquisition and reconsolidation of a cocaine-induced conditioned place preference memory. J Neurosci 35:4190–4202. 10.1523/JNEUROSCI.3592-14.2015 25762666 PMC4355195

[B51] Slaker ML, Harkness JH, Sorg BA (2016) A standardized and automated method of perineuronal net analysis using Wisteria floribunda agglutinin staining intensity. IBRO Rep 1:54–60. 10.1016/j.ibror.2016.10.001 28713865 PMC5507617

[B52] Sleipness EP, Sorg BA, Jansen HT (2005) Time of day alters long-term sensitization to cocaine in rats. Brain Res 1065:132–137. 10.1016/j.brainres.2005.10.017 16309631

[B53] Sohal VS, Zhang F, Yizhar O, Deisseroth K (2009) Parvalbumin neurons and gamma rhythms enhance cortical circuit performance. Nature 459:698–702. 10.1038/nature07991 19396159 PMC3969859

[B54] Sparta DR, Hovelso N, Mason AO, Kantak PA, Ung RL, Decot HK, Stuber GD (2014) Activation of prefrontal cortical parvalbumin interneurons facilitates extinction of reward-seeking behavior. J Neurosci 34:3699–3705. 10.1523/JNEUROSCI.0235-13.2014 24599468 PMC3942585

[B55] Suttkus A, Rohn S, Weigel S, Glöckner P, Arendt T, Morawski M (2014) Aggrecan, link protein and tenascin-R are essential components of the perineuronal net to protect neurons against iron-induced oxidative stress. Cell Death Dis 5:e1119. 10.1038/cddis.2014.25 24625978 PMC3973247

[B56] Van den Oever MC, Spijker S, Smit AB, De Vries TJ (2010a) Prefrontal cortex plasticity mechanisms in drug seeking and relapse. Neurosci Biobehav Rev 35:276–284. 10.1016/j.neubiorev.2009.11.016 19932711

[B57] Van den Oever MC, Lubbers BR, Goriounova NA, Li KW, Van der Schors RC, Loos M, Riga D, Wiskerke J, Binnekade R, Stegeman M, Schoffelmeer AN, Mansvelder HD, Smit AB, De Vries TJ, Spijker S (2010b) Extracellular matrix plasticity and GABAergic inhibition of prefrontal cortex pyramidal cells facilitates relapse to heroin seeking. Neuropsychopharmacology 35:2120–2133. 10.1038/npp.2010.90 20592718 PMC3055295

[B58] Vazquez-Sanroman D, Leto K, Cerezo-Garcia M, Carbo-Gas M, Sanchis-Segura C, Carulli D, Rossi F, Miquel M (2015a) The cerebellum on cocaine: plasticity and metaplasticity. Addict Biol 20:941–955. 10.1111/adb.12223 25619460

[B59] Vazquez-Sanroman D, Carbo-Gas M, Leto K, Cerezo-Garcia M, Gil-Miravet I, Sanchis-Segura C, Carulli D, Rossi F, Miquel M (2015b) Cocaine-induced plasticity in the cerebellum of sensitised mice. Psychopharmacology 232:4455–4467. 10.1007/s00213-015-4072-1 26482898

[B60] Vazquez-Sanroman DB, Monje RD, Bardo MT (2017) Nicotine self-administration remodels perineuronal nets in ventral tegmental area and orbitofrontal cortex in adult male rats. Addict Biol 22:1743–1755. 10.1111/adb.12437 27549591 PMC5322253

[B61] Vo T, Carulli D, Ehlert EM, Kwok JC, Dick G, Mecollari V, Moloney EB, Neufeld G, de Winter F, Fawcett JW, Verhaagen J (2013) The chemorepulsive axon guidance protein semaphorin3A is a constituent of perineuronal nets in the adult rodent brain. Mol Cell Neurosci 56:186–200. 10.1016/j.mcn.2013.04.009 23665579

[B62] Wang D, Fawcett J (2012) The perineuronal net and the control of CNS plasticity. Cell Tissue Res 349:147–160. 10.1007/s00441-012-1375-y 22437874

[B63] Womelsdorf T, Schoffelen JM, Oostenveld R, Singer W, Desimone R, Engel AK, Fries P (2007) Modulation of neuronal interactions through neuronal synchronization. Science 316:1609–1612. 10.1126/science.1139597 17569862

[B64] Xue Y-X, Xue L-F, Liu J-F, He J, Deng J-H, Sun S-C, Han H-B, Luo Y-X, Xu L-Z, Wu P (2014) Depletion of perineuronal nets in the amygdala to enhance the erasure of drug memories. J Neurosci 34:6647–6658. 10.1523/JNEUROSCI.5390-13.2014 24806690 PMC6608143

[B65] Yamada J, Ohgomori T, Jinno S (2015) Perineuronal nets affect parvalbumin expression in GABAergic neurons of the mouse hippocampus. Eur J Neurosci 41:368–378. 25411016 10.1111/ejn.12792

