# Supplementary material for: Cocaine Exposure Modulates Perineuronal Nets and Synaptic Excitability of Fast-Spiking Interneurons in the Medial Prefrontal Cortex
Source: eNeuro. 2018 Oct 4;5(5):ENEURO.0221-18.2018. doi: 10.1523/ENEURO.0221-18.2018 (PMC6171740; doi:10.1523/ENEURO.0221-18.2018)
Supplement: Figure 1-1 — Table showing significant correlations between cocaine-induced locomotor activity and WFA or PV intensity in single- and double-labeled cells in the prelimbic and infralimbic PFC. Download Figure 1-1, DOC file. [file sup_enu-eN-NWR-0221-18-s01.doc]

| **Group** | **Region** | **Day of treatment** | **WFA^+^/PV^+^** (p, R^2^, slope) | **WFA^+^/PV^-^** (p, R^2^, slope) |
| --- | --- | --- | --- | --- |
| 1 Day, 24 h | Infralimbic | Day 1 | p = 0.0146, R^2^ = 0.8092, neg | p = 0.0267, R^2^ = 0.7457, neg |
| 5 Days, 2 h | Prelimbic | Day 2 | p < 0.0001, R^2^ = 0.9576, pos ***** | p = 0.0138, R^2^ = 0.6634, pos |
|  |  | Day 3 | p = 0.0032, R^2^ = 0.7898, pos | p = 0.0430, R^2^ = 0.5218, pos |
|  | Infralimbic | Day 5 | -------- | p = 0.0125, R^2^ = 0.6742, pos |
| 5 Days, 24 h | Prelimbic | Day 1 | -------- | p = 0.0462, R^2^ = 0.6705, neg |
|  | Infralimbic | Day 3 | p = 0.0363, R^2^ = 0.7057, pos | p = 0.0170, R^2^ = 0.7947, pos |
|  |  |  | **PV^+^/WFA^+^** | **PV^+^/WFA^-^** |
|  | Prelimbic | Day 1 | p = 0.0124, R^2^ = 0.8237, neg | p = 0.0364, R^2^ = 0.7056, neg |

**Figure 1-1.** Correlation between cocaine-induced locomotor activity and WFA or PV intensity

***** Shown in Figure 1D
